# Our Platform

**Published:** 1871-10

**Authors:** 


### OUR PLATFORM.

The following extract is taken from a letter received from one of the Associate Editors, a personal friend of the Senior Editor. Our Associate says:

"The more I think of it, the more I feel there is a necessity for a full and forcible setting forth of your purposes and intentions in regard to your journal—a clear and distinct statement of the moral and professional duties and responsibilities of the Associate Editors. Plainly, all whose services you wish to enlist, have not the same advantages that I have, of a long personal acquaintance with you."

We are pleased to have the opportunity to *plainly* state "Our Position" and what we conceive to be the "*moral*" as well as

"professional" responsibilities of our Associate Editors. THE COMPANION, in its "heart of hearts," is in full sympathy and accord with the very highest moral principles underlying the "good, beautiful and true," in man. We believe no substantial character can be erected upon any other basis than that of the very purest platform of morals. The highest type of manhood—of humanity beautified—is *that* which is embodied in the "golden rule," which, as an epitome of the whole moral law, came from God, through "The Word"—in Divine simplicity flashing the grand truth over land and sea "Do to others as you would that they should do to you." Upon this "rock" is built every moral edifice—without such a foundation every other is worthless and deceptive. Upon this is reared the beautiful moral principles contained in the Code of Medical Ethics. Were every physician a Christian—not in name only, but in word and deed: not a hearer of the law, but a doer of it—there would be no necessity for a code of medical ethics. The moral law was instituted not for the righteous but to restrain the evil, and to vindicate the truth. As all men, without a single exception, are "sinners," and hence unrighteous, it became necessary to invoke the moral law, as a "School-Master," to lead them to the higher, nobler, principle of Faith in One who, for man, should keep the law. In medical ethics there is not one requisition at war with the principles of this moral law, viewed in its very lowest aspect. To be kind, courteous, truthful, honest, generous—in other words, to be, in word and act, at all times and under all circumstances the Christian gentleman, is the spirit and teachings of the code. In such a "system" of ethics there is no room for the charlatan, or the medical demagogue; not an "inch of space" for the traducer or slanderer. In such a system—the defamer of professional reputation and character, is an outsider; within its pure boundaries no medical "scalawag" or unprofessional trickster, can be citizenized. These are the views—these the glorious principles which it becomes the delightful pleasure and duty of THE COMPANION to support, advocate, and defend. If any man, or set of men, "teach any other doctrine" and call it ethics; or are governed and controlled by principles other than those embodied in the Christian's code, he is a stranger and a foreigner; a poor hypocrite, unworthy even the frowns of a good man's contempt.

This is "our platform." On its beautiful surface there is not a plank upon which such characters may stand—not one under which deceit, fraud and slander may hide. Such a platform cannot be successfully used as a theatre for the tricks and rings of medical shoddyites, and one upon which they may practice the "Black Arts."

Upon such a plantform we *know* our Associates stand. We would scorn to ask an honest man to become "an Associate" upon any other. If we laud the ethics, it is for the glory of medicine; if we support the truth, it is that error may be throttled and principle exalted. In such a contest we *count* all good men;—we ask their support and endorsement only in the Right. As for the rest—the wirery, the slippery, the "non-commitalist," the demagogue—we give "them a wide berth and an open sea."

As to the duties of our "Associates," in regard to THE COMPANION, they must largely determine for themselves, under the impetus of doing their best for the honor of medicine and the interests of the Profession. We invite them to labor together for the common good—to write not alone upon medicine, but upon all topics legitimately allied to it, by which it may be honored and respected. In so noble a work let all aim at grand results in everything pertaining to our glorious calling.

